# Enhancing Psychophysiological Well‐Being Through Nature‐Based Soundscapes: An Examination of Heart Rate Variability in a Cross‐Over Study

**DOI:** 10.1111/psyp.14760

**Published:** 2025-01-13

**Authors:** Susanne Kumpulainen, Samad Esmaeilzadeh, Markus Pesonen, Catarina Brazão, Arto J. Pesola

**Affiliations:** ^1^ Active Life Lab South‐Eastern Finland University of Applied Sciences Mikkeli Finland; ^2^ Olo Space Oy Ihamaniemi Finland

**Keywords:** heart rate variability, music, nature, soundscape, therapy, well‐being

## Abstract

Stress and psychological disorders are substantial public health concerns, necessitating innovative therapeutic strategies. This study investigated the psychophysiological benefits of nature‐based soundscapes, drawing on the biophilia hypothesis. Using a randomized, acute cross‐over design, 53 healthy participants experienced either a nature‐based or a reference soundscape for 10 min, with a 2‐min washout period. The nature‐based soundscape integrated nature sounds with elements of music to create an immersive nature experience. A calm coffee shop soundscape without discernible speech was selected as a reference to represent a typical urban relaxation environment. Heart rate variability (HRV) was the primary outcome, with exploratory outcomes including heart and respiratory rates, and questionnaires assessing affective well‐being, creativity, and belonging. Results showed that the nature‐based soundscape significantly improved HRV and reduced heart and respiratory rates, indicating enhanced parasympathetic activity. Participants reported lower feelings of anxiety and depression and increased feelings of comfort, enthusiasm, creativity, and belonging. This study highlights the multifaceted benefits of nature‐based soundscapes, suggesting they could serve as easily accessible therapeutic options for promoting immediate recovery and reducing daily stress in healthy individuals.

## Abstract 2

To our knowledge, this is the first comprehensive demonstration of the physiological and psychological relaxation benefits derived from nature‐based soundscapes. The soundscape, combining a variety of nature sounds with musical elements, increased heart rate variability and decreased both heart and respiratory rates, alongside improved subjective evaluations in emotional, cognitive, and social domains. This highlights the potential of nature‐based soundscapes as an accessible therapeutic method.

## 1 Health Benefits of Natural Environments

Psychological distress and mental health disorders have become critical public health challenges in modern society (Gruber et al. 2021). The rising prevalence of stress, anxiety, and depression affects individuals across all age groups, leading to an augmented influx of admissions related to mental health issues within healthcare systems (Gruber et al. 2021; Lind et al. 2023). Contributing factors like the COVID‐19 pandemic and growing social media usage have intensified social isolation and avoidance of crowded spaces (Brooks et al. 2020; Primack et al. 2017). Epidemiological research indicates that psychiatric disorders, such as mood and anxiety conditions, are more common in urban settings compared to rural ones suggesting that urbanization and reduced contact with nature may play a role (Costa e Silva and Steffen 2019; Davis et al. 2021; Krabbendam and van Os 2005; Peen and Dekker 2004; Xu et al. 2023). In response to these escalating mental health demands, both scientific research and practical applications are imperative (Gruber et al. 2021).

Historically, nature has played a pivotal role in aiding human recovery from psychophysiological stress (Lavelle Sachs et al. 2024; Maller et al. 2006; White et al. 2023). Central to this understanding is the well‐known biophilia hypothesis, which postulates that humans possess an inherent affiliation to connect with the natural environment (Wilson 1984). The well‐being benefits of nature have been extensively researched, with two predominant theoretical frameworks connected to the biophilia hypothesis: the attention restoration theory (ART) and the stress recovery theory (SRT). Introduced by Kaplan and Kaplan 1989, ART examines the revitalizing effects of nature on depleted attentional resources. On the other hand, SRT, proposed by Ulrich 1983, emphasizes the restorative power of natural settings in alleviating stress. SRT establishes a more direct association between nature exposure and physiological shifts in autonomic balance toward parasympathetic “rest‐digest” activation, with a simultaneous reduction in sympathetic “fight‐flight” activation.

These theories have support from research demonstrating links between nature exposure and enhanced physiological and psychological well‐being (Berto 2014; Bratman et al. 2019; Haluza, Schönbauer, and Cervinka 2014; Mygind et al. 2019). Urbanization, time constraints, and disabilities, among other barriers, often impede access to real nature. Such barriers highlight the pressing need to explore technological innovations capable of simulating natural environments, ensuring accessibility for all. Acoustic experiences of nature are increasingly identified as important ecosystem services that facilitate psychophysiological restoration (Francis et al. 2017; Kong and Han 2024). Upon exposure to nature soundscapes, individuals might conjure personalized visualizations of natural environments (Bates et al. 2020; Ratcliffe 2021), an experience readily accessible via speakers or headphones.

### 1.1 Previous Studies on Nature Soundscapes

A soundscape can emanate from an individual sound or a combination of sounds originating from an engaging environment. For the purpose of this discussion, a soundscape is defined as an individual's perception of the acoustic environment within a specific context (Pijanowski et al. 2011). An increasing number of psychological studies show perceived restorative experiences from nature soundscapes, including feelings of pleasure, relaxation, and a reprieve from daily stresses (Buxton et al. 2021; Erfanian et al. 2019; Ratcliffe 2021). Sounds like birdsong, wind, and water are frequently associated with serene natural settings. The combination of natural sounds reflecting biodiversity is often found to be more pleasing than the sound of a single species (Fisher et al. 2021; Hedblom et al. 2014). Nonetheless, limited evidence currently supports the physiological well‐being effects of nature soundscapes (Erfanian et al. 2019).

Nature virtual reality paired with sound, as opposed to without sound, amplifies physiological responses (Annerstedt et al. 2013; Naef et al. 2022; Ojala et al. 2022). However, sound alone did not produce relaxing effects in these studies (Naef et al. 2022; Ojala et al. 2022). Several studies have contrasted the effects of natural soundscapes with traffic noises (Alvarsson, Wiens, and Nilsson 2010; Hedblom et al. 2019; Jo et al. 2019; Medvedev, Shepherd, and Hautus 2015). Some of these studies found that natural sounds contributed to improved recovery metrics, such as decreased skin conductance, heart rate (HR), and oxy‐Hb concentrations in the prefrontal cortex (Alvarsson, Wiens, and Nilsson 2010; Jo et al. 2019). However, within these studies and others, no significant differences in skin conductance or heart rate variability (HRV) were reported (Alvarsson, Wiens, and Nilsson 2010; Hedblom et al. 2019; Medvedev, Shepherd, and Hautus 2015). These mixed results may be influenced by various factors, including differences in experimental design or participant characteristics. Whereas the contrast of nature sounds and traffic noises does illuminate the stark differences between the two soundscapes, it may not provide the most informative insight into the relaxation potential of nature sounds. Traffic noises, for many individuals, are intrinsically associated with stress and discomfort (Buxton et al. 2021), serving as a less‐than‐ideal baseline for relaxation. Using a condition that elicits stress as a reference might only establish that nature sounds are less stressful than traffic noises, rather than essentially relaxing.

Gould van Praag et al. (2017) investigated the differences between artificial and naturalistic soundscapes. While they did not identify significant distinctions in default brain activity, they noted a functional connectivity shift from the anterior to the posterior midline when participants were exposed to the naturalistic soundscape. This connectivity shift aligns more closely with SRT rather than ART, as posterior brain regions have been associated with parasympathetic activity and relaxation, while anterior regions, such as the medial prefrontal cortex, are linked to sympathetic arousal and self‐referential processing (Beissner et al. 2013). The study did not find evidence of increased default mode network activity in the naturalistic condition, which would be expected under ART, but rather observed shifts in autonomic balance, a finding consistent with SRT.

In a subsequent study, Qi et al. (2022) evaluated the physiological responses of individuals listening to bird songs, compared to a control group exposed to an environment without bird songs. The results indicated that those in the bird song group exhibited lowered skin conductance levels and heightened alpha brain activity, which are markers of relaxation. Nonetheless, several studies did not find significant physiological differences when comparing natural soundscapes to various conditions: silence (Ghezeljeh et al. 2017; Largo‐Wight, O'Hara, and Chen 2016), classical music (Largo‐Wight, O'Hara, and Chen 2016), or several different samples including traffic and building construction sounds (Medvedev, Shepherd, and Hautus 2015). While there is a well‐established connection between nature soundscapes and psychological restoration, the evidence concerning physiological effects remains sparse. It seems that pure nature sounds might not be sufficiently effective in inducing physiological changes, especially when compared to more neutral references. To form a more comprehensive understanding of nature sounds' potential as relaxation aids, further high‐quality cross‐over research, incorporating appropriate control conditions, is essential (Erfanian et al. 2019; Ratcliffe 2021).

### 1.2 Combination of Nature Sounds and Music

Music research can inform designing nature‐based soundscapes with the aim to improve efficacy beyond plain nature sounds. Music is created by ordering tones or sounds in succession, in combination, and in temporal relationships to produce a composition having unity and continuity (Murrock and Higgins 2009). An expanding body of scientific research is showing the positive impacts of musical auditory stimulation on various physiological outcomes including blood pressure, HR, respiration rate, electrothermal activity and biochemical indicators (Chafin et al. 2004; Koelsch and Jäncke 2015; Kulinski et al. 2022; Mojtabavi et al. 2020). Across various populations, music consistently appears to have a positive impact on parameters related to HRV (Kulinski et al. 2022; Mojtabavi et al. 2020). The potential of music as a non‐pharmacological therapeutic intervention is gaining attention, with research indicating its capacity to enhance health outcomes in both healthy individuals and those with medical conditions, such as depression and anxiety (Mojtabavi et al. 2020).

Different musical genres can elicit varying emotional and physiological responses. Tranquil music might promote relaxation, while rhythmic tunes can heighten arousal (Chafin et al. 2004; Koelsch and Jäncke 2015). However, the physiological and emotional effects of music extend beyond genre. Factors such as personal preferences, individual interpretation, and current mood state play pivotal roles (Koelsch and Jäncke 2015; Kulinski et al. 2022). For instance, research employing a uniform musical piece for all participants often yielded inconsistent outcomes due to these individual variations (Mojtabavi et al. 2020). Music that aligns with individual preferences tends to enhance desired outcomes. For instance, preferred music has been shown to improve post‐exercise recovery (Jia et al. 2016). Similarly, better pain control was experienced by fibromyalgia patients when they liked the therapeutic music (Linnemann et al. 2015). Moreover, it has been demonstrated that sounds perceived as pleasant resulted in lower skin conductance levels during rest compared to unpleasant sounds. This suggests that the autonomic function during relaxation can be influenced by the subjective response to the acoustic environment (Medvedev, Shepherd, and Hautus 2015). A significant limitation in current research is the inadequate control for individual differences in musical preference and perception (Mojtabavi et al. 2020). Given that a single musical track can elicit diverse reactions both in intensity and nature, it is challenging to identify one specific type of music as universally effective for therapeutic relaxation.

Both music and nature sounds have demonstrated significant potential as therapeutic mediums. However, the subjective resonance of music, influenced by personal preferences, may impact its universal applicability. Similarly, plain nature sounds may not induce strong enough physiological responses to be fully effective as a standalone intervention, even though the physiological effects of music have been more consistently demonstrated. The combination of nature sounds with musical elements, however, holds potential for amplifying and complementing the benefits these auditory stimuli offer individually. This combined approach could provide a more immersive and effective means of relaxation and restoration by engaging the listener's emotional and physiological systems on a deeper level. In integrating the structured properties of music with the biophilic qualities of natural soundscapes, this method may bridge the gap between preference‐driven responses to music and the intrinsic soothing qualities of nature, enhancing overall well‐being.

The combination of music and nature sounds has been studied in hospital settings, where it was found to reduce pain sensation, anxiety, and enhance relaxation in patients (Amiri et al. 2017; Bauer et al. 2011). Furthermore, the effects of nature‐based music on several HRV parameters with a between‐subject study design during dental procedures have been demonstrated (Wang et al. 2016). However, these effects were not prevalent in known parasympathetic parameters but instead, they were observed, for example, in the parameter of low‐frequency to high‐frequency ratio (LF/HF), the physiological underpinnings of which remain unclear (Laborde, Mosley, and Thayer 2017; Shaffer and Ginsberg 2017). These findings suggest that the combination of nature sounds with music could be of significant therapeutic value for relaxation and restoration, in alignment with SRT, providing a structured framework. However, the need for rigorous research to objectively demonstrate these effects remains a critical aspect of this field.

### 1.3 Psychophysiology of Auditory Stimulation in Combining Nature Sounds With Musical Elements

From a psychophysiological perspective, sounds are detectable signals in our external environment that can elicit a series of unconscious responses. Physiological responses are regulated by the autonomic nervous system (ANS), which plays a crucial role in modulating inherent bodily functions to maintain homeostasis and adapt to external and internal conditions. Specific neural pathways are responsible for transmitting auditory signals, and various brain regions are implicated in the processing of these stimuli (Irwin et al. 2011).

The ascending auditory pathway, extending from the cochlea to the auditory cortex, is crucial for responding to the physical and acoustic properties of sound, and it processes these properties irrespective of the emotional content of the sound (Irwin et al. 2011). Conversely, areas within the limbic system, such as the amygdala and the insula, play significant roles in processing the emotional valence of auditory stimuli and are part of the central nervous system's response to auditory information (Frühholz, Trost, and Grandjean 2014). The integration of auditory information from both these ascending pathways shapes the perception (Irwin et al. 2011). Consequently, the ANS's response stems from a combination of bottom‐up and top‐down processes. Individual factors—including personal preferences, heritage, past experiences, current life situations, and circumstances—specifically influence the semantic properties and emotional response of an auditory stimulus (Ratcliffe 2021). As a result, ANS responses can vary among individuals exposed to the same music and even fluctuate within an individual at different time points.

The biophilia hypothesis suggests humans have an innate affinity for nature, possibly rooted in evolutionary advantages related to survival and reproduction (Wilson 1984). This affinity might be influenced by the limbic system, an older component of the brain integral to unconscious emotional processing (Roxo et al. 2011; Sudimac, Sale, and Kühn 2022). Considering how our ancestors responded to natural stimuli, such as the sound of flowing water indicating a nearby stream or the sight of greenery signifying food sources, it becomes apparent that reacting positively to these cues would have been evolutionarily beneficial. Consequently, nature‐based soundscapes that stimulate the limbic system may offer more consistent beneficial emotional responses than traditional music (Frühholz, Trost, and Grandjean 2014; Irwin et al. 2011).

### 1.4 The Present Study

The nature‐based soundscape utilized in this study integrated a variety of nature sounds with elements of music, creating an acoustic nature experience having by unity and continuity. This combination aimed to amplify the immersive quality of the soundscape and enhance its efficacy in inducing relaxation. The calm coffee shop soundscape was selected as a reference condition to represent a typical urban relaxation environment, thereby providing a comparative benchmark (Ferreira, Ferreira, and Bos 2021; Lee 2022). Coffee shops, recognized as ‘third places,’ play a pivotal role in enhancing quality of life by offering spaces conducive to rest, escape from daily routines, socialization, and emotional release (Lee 2022).

The purpose of this study was to compare the psychophysiological effects of the nature‐based and reference soundscapes in healthy individuals to see the difference in relaxation between natural and urban environments. If effective in inducing well‐being benefits, nature‐based soundscapes could serve as a preventive approach for managing mental health by reducing daily stress. Based on the biophilia hypothesis, we expected that the nature‐based soundscape would yield more consistent beneficial physiological responses due to the human brain's innate positive reaction to natural elements.

Responses to auditory stimuli were measured with HRV, which is a noninvasive and well‐established indicator of overall psychophysiological well‐being. The root mean square of successive differences (RMSSD), a specific HRV parameter, represents parasympathetic activity and is a recommended metric in psychophysiological evaluations (Laborde, Mosley, and Thayer 2017). We hypothesized that RMSSD, as the primary outcome, would be higher during exposure to a nature‐based soundscape than in the reference condition, supporting SRT. Additionally, as explorative secondary objectives, we analyzed HR, low frequency (LF), high frequency (HF) and respiratory rate as well as administered questionnaires to assess affective well‐being (Warr 1990), creativity, and a sense of belonging using visual analog scale (VAS) (Hayes and Patterson 1921).

## 2 Materials and Methods

### 2.1 Participants

In the study, 53 healthy volunteers were recruited, of whom 37 identified as women, 16 as men, and none as other. Participants represented multiple nationalities, including Finnish, Australian, Afghan, Lithuanian, Brazilian, and Russian backgrounds. Recruitment was conducted in both English and Finnish through digital platforms, including Twitter, LinkedIn, Instagram, and Facebook, to ensure a wide and diverse pool of participants. Questionnaires were administered in Finnish or English, depending on the participants' language preference, to ensure comprehension and accurate responses. The participants had a mean age of 39 years (SD = 9), a height of 167 cm (SD = 25) and a weight of 75 kg (SD = 15). Participants represented a wide range of occupations, including massage therapists, managers, researchers, entrepreneurs, instructors, nurses, family workers, mechanics, and secretaries, reflecting diverse professional fields. This diversity indicates a well‐rounded participant group, representing sectors such as healthcare, education, business, and freelance work. The study was advertised as a “Break study,” deliberately omitting any reference to nature or its potential well‐being effects to prevent influencing participants' attitudes or the outcomes. Eligibility criteria excluded individuals with cardiovascular conditions, arrhythmia, or those on medications known to affect heart rate variability. Prior to participating, individuals were instructed to abstain from exercise and alcohol for 24 h and avoid consuming food, caffeine, smoking and other stimulants for 2 h preceding the measurements. All participants received a detailed overview of the study procedures and provided written informed consent.

Based on the RMSSD results from Gladwell et al. (2012) and an anticipated minimum effect size of 0.40, it was determined that 45 participants would provide 80% power at a significance level of 0.05 using a two‐tailed test, assuming an *r* correlation of 0.90 between the two measurements, given that both were taken within a 30‐min window. For secondary outcomes, the minimum detectable differences with 80% power at a significance level of 0.05 were determined as follows: mean heart rate (1.8 bpm), LF (600 ms^2^/Hz), HF (160 ms^2^/Hz), respiratory rate (0.5 breaths/min), affective well‐being (0.19), belonging (0.29) and creativity (0.85). Calculations for affective well‐being were derived from Virtanen et al. (2021), for belonging from Cheryan, Meltzoff, and Kim (2011), and for creativity from Douglas et al. (2022). To account for potential data loss due to unforeseen issues, such as equipment malfunctions or participant withdrawals, we recruited an additional eight participants. Due to technical issues, such as the unsuccessful saving of data, HRV data could not be analyzed from two participants.

Ethical approval for the study was granted by the Ethics Committee of South‐Eastern Finland University of Applied Sciences, and the research was conducted in alignment with the principles of the Declaration of Helsinki.

### 2.2 Soundscape Interventions

The crafted nature‐based soundscape (Olo Space Oy, Ihamaniemi, Finland) features binaural rendering of authentic recordings from the European Lakeland region combined with minimalistic music sequences (available at https://on.soundcloud.com/sf8tTou76DWh6NeZ6). The instrumentation incorporates water, birds, insects, wind, thunder, gentle rain, campfire, bells, spacious drone (a deep, sustained sound), and a sparse melody played on a Portuguese guitar. The diverse collection of nature sounds was integrated with elements of music to create an immersive acoustic nature experience having unity and continuity. The goal is to provide a realistic and immersive experience of nature that is accessible even within the confines of modern urban life.

This approach acknowledges the challenges posed by contemporary living environments and offers a method to reconnect with the natural world. As a reference, the soundscape used is an ambient recording of a calm coffee shop with indistinct spoken language (available at: https://on.soundcloud.com/Tiu1D1tfFj3Kq1yUA). This choice is informed by its recognized association with relaxation and leisure in urban settings, providing a baseline benchmark for comparison (Ferreira, Ferreira, and Bos 2021). Coffee shops have been demonstrated to offer psychological benefits similar to urban parks, providing a pleasant space for rest (Lee 2022). Furthermore, a study in a restaurant context revealed that participants experienced greater pleasantness when exposed to cafeteria sounds compared to silence, further supporting the relevance of this choice of ambient sound for our study (Mathiesen et al. 2022).

Both the nature‐based and coffee shop conditions allow mind to wander without demanding focused cognitive engagement. Each audio track was 10 min long, with a quality of 16‐bit, 44.1 kHz. Sound samples were processed, and their loudness levels matched using Ableton Live to ensure any observed effects were attributed to the sound content rather than volume differences. This normalization process involved adjusting each sample to achieve consistent loudness levels, measured in LUFS (Loudness Units relative to Full Scale). The loudness of each sample was verified using “Youlean Loudness Meter 2,” with the nature‐based soundscape measured at −32.4 LUFS and the coffee shop sound at −32.3 LUFS. Playback was facilitated through headphones (QuietComfort, Bose Corporation, Framingham, USA). Participants were provided with a blindfold to eliminate visual stimuli.

### 2.3 Experimental Design

**FIGURE 1:**
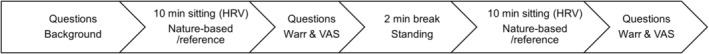
Experiment Timeline. Measurements were carried out in a single occasion for each participant. The order of exposure to the nature‐based and the reference soundscapes was randomized to control for sequence effects.

**FIGURE 2:**
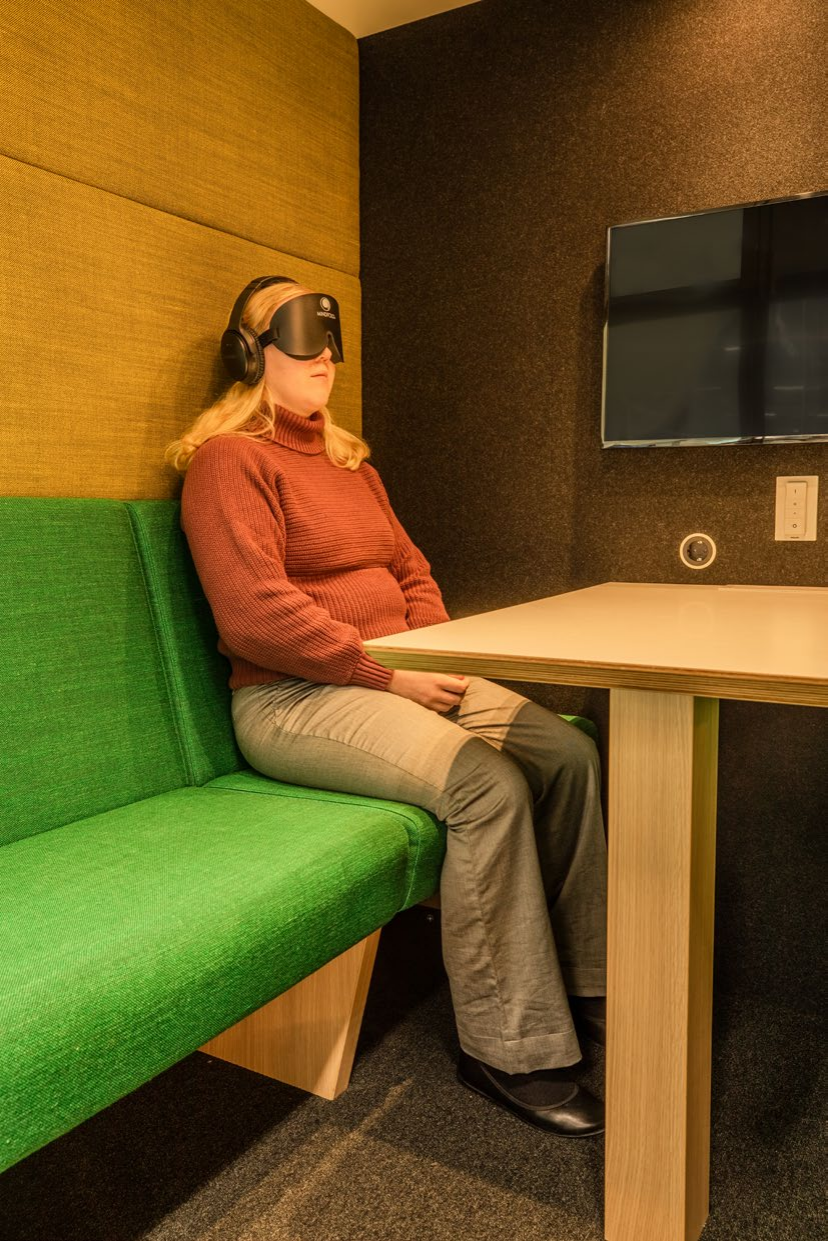
Photo of the measurement setup. Photograph by Manu Eloaho.

Participants underwent measurements on a single occasion, during which they listened to both nature‐based and reference soundscape in a randomized order (Urbaniak and Plous 2013). The procedures for both soundscape sessions were identical. The sequence of events within the experiment is depicted in Figure 1. First, participants filled out a background information questionnaire, where we asked them about their gender, age, height, weight, and professional background. Following this, participants sat and relaxed for 10 min in a quiet private room (SmartMeeting Block, Smartblock Oy, Helsinki, Finland) as they listened to one of the soundscapes (Figure 2). During this time, the glass door remained closed, and the researcher monitored their HRV from outside. After each listening session, participants were handed an iPad to complete the Warr's affective well‐being and VAS questionnaires. There was a 2‐min washout period outside the room during which participants stood before starting the second listening session (Alvarsson, Wiens, and Nilsson 2010; Li and Kang 2019).

### 2.4 HRV Measurement

We used the Polar H10 heart rate sensor (Polar Electro, Kempele, Finland), which has a 1000‐Hz sampling frequency, to record HRV data. The Polar H10 is an advanced model of the Polar H7, which has been shown to be a valid heart rate monitor when recording beat‐to‐beat (RR) intervals for HRV analyses (Giles, Draper, and Neil 2016). Participants wore the sensor affixed with a chest strap at the xiphoid process level. The sensor was paired via Bluetooth with the Kubios HRV application on an iPad. During the 10‐min measurement period, participants remained seated. We allocated the first 5 min for heart rate stabilization and analyzed the data from the subsequent 5 min.

The Kubios HRV Scientific 4.0.1 software (Niskanen et al. 2004) processed the raw HRV data, which was stored as RR interval data files. We identified and corrected artifacts in line with international Task Force guidelines (Malik 1996) using a verified automatic correction method (Lipponen and Tarvainen 2019). After correction, we referred to the RR interval data as normal‐to‐normal (NN) data. We then analyzed several outcomes: the primary outcome of RMSSD and secondary outcomes including HR, LF, HF, and respiratory rate, providing a comprehensive description as recommended by Laborde, Mosley, and Thayer (2017). We derived the respiratory rate estimation from the RR data using the Kubios software (Tarvainen et al. 2014).

We employed two resting state HRV measurements to compare differences between conditions, as outlined in the study which informed our sample calculation (Gladwell et al. 2012). According to HRV guidelines, short‐term resting HRV recordings should last at least 5 min and be conducted following a stabilization period of no < 5 min (Malik et al. 1996; Laborde, Mosley, and Thayer 2017). Our study utilized an acute randomized crossover design, which allows for direct comparison between two conditions, each serving as its own baseline (Gladwell et al. 2012).

### 2.5 Psychological Measurement

We utilized Warr's questionnaire (Warr 1990) to measure affective well‐being, an instrument grounded in the circumplex model of affect by Russell (1978). Given their comprehensive nature, dimensional models are suitable for depicting fundamental affective reactions. Affective well‐being has frequently been used as an indicator of internal recovery in prior research (Kim, Park, and Niu 2017; Virtanen et al. 2021). Warr's 12‐item scale encompasses six positive emotions (calm, contented, relaxed, cheerful, enthusiastic, optimistic) and six negative ones (tense, uneasy, worried, depressed, gloomy, miserable). It is designed to measure dimensions of anxious–comfort (equivalent to the unpleasant high activation—pleasant low activation dimension) and depressed–enthusiastic (equivalent to the pleasant high activation—unpleasant low activation dimension), providing a comprehensive affective result. Participants evaluated their immediate emotional state on a 10‐point sliding scale with one decimal precision, where 0 signifies “not at all” and 10 represents “extremely”. In the analysis of these assessments, we adopted a four‐factor solution, categorizing self‐reported emotions into anxiety (Cronbach's *α* = 0.82), comfort (Cronbach's *α* = 0.91), depression (Cronbach's *α* = 0.83), and enthusiasm (Cronbach's *α* = 0.81), following the methodology recommended by Mäkikangas, Feldt, and Kinnunen (2007). It is important to note that Warr's questionnaire is designed to assess self‐reported feelings related to emotional well‐being and is not intended for the diagnosis or treatment of medical conditions.

To shed some light on cognitive and social well‐being aspects, we queried participants about their feelings of creativity and belonging (Kumpulainen, Esmaeilzadeh, and Pesola 2024). Creativity, considered a cognitive function, was assessed to determine whether nature‐based soundscapes support the assumptions of the ART. Creativity is classified as a cognitive function because it involves complex mental processes such as problem‐solving, idea generation, and making novel connections between concepts (Dietrich 2004). The feeling of belonging was evaluated to gauge its impact on social well‐being. Participants' responses were captured using VAS (Hayes and Patterson 1921), which ranged from 0 to 10 (with one decimal precision). On this scale, 0 represented “not at all,” while 10 signified “extremely”. The specific questions posed to participants were: “How would you rate your creativity at the moment?” (Creativity) and “How much do you feel connected to other people right now?” (Belonging). While the VAS is a validated tool for capturing subjective, momentary states and experiences (Aitken 1969; Escalona‐Marfil et al. 2020), the use of VAS for measuring creativity and belonging in this study is exploratory, as these specific constructs are not validated. The single‐item questions were used to capture participants' immediate subjective feelings, acknowledging that they may not represent validated multi‐item scales and the results for these constructs should therefore be interpreted with caution.

### 2.6 Statistical Analysis

The Shapiro–Wilk test was used to determine the normality of the data distribution. Variables that adhered to normality assumptions, including heart rate, respiratory rate, and enthusiasm, were analyzed using the paired *t*‐test to compare conditions (nature‐based soundscape vs. reference soundscape). Variables that did not meet normality assumptions were analyzed using the robust paired *t*‐test (Wilcox 2016; Yuen 1974). We used the WRS2 package (Mair and Wilcox 2020) for R (R Core Team 2021) to conduct the analysis. For the paired *t*‐test, effect size is reported as Cohen's *d*, and for the robust paired *t*‐test, the explanatory effect size (*d*) is provided (Algina et al. 2005; Cohen 1988).

## 3 Results

### 3.1 Physiological Parameters

**FIGURE 3:**
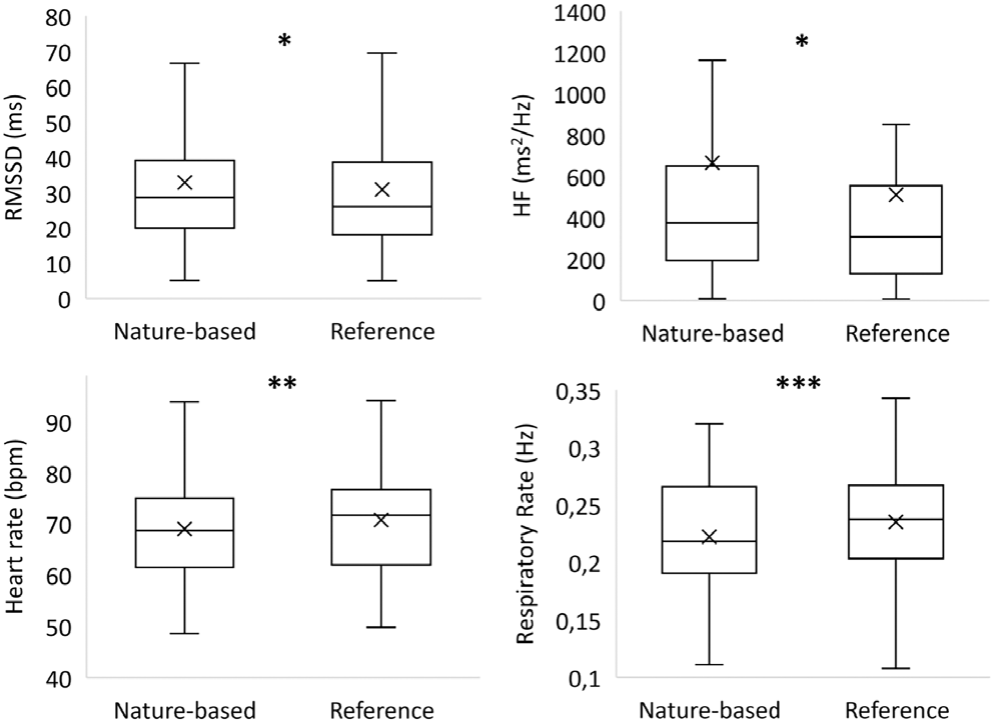
Results for the root mean square of successive differences (RMSSD), high frequency (HF) heart rate and respiratory rate. Given the non‐normal nature of the HRV data, the distribution is depicted using box plots. The middle line of the box represents the median, while an “x” inside the box represents the mean. The bottom and top lines of the box represent the interquartile range, and vertical lines extending from the ends of the box indicate variability outside the upper and lower quartiles. Significance levels for the statistical tests are denoted as follows: *p < 0.05, **p < 0.01, and ***p < 0.001.

The physiological outcome values are presented in Table 1. Consistent with our primary hypothesis, the time‐domain measure RMSSD was significantly higher during the nature‐based soundscape compared to the reference soundscape, *t*(30) = 2.06, *p* = 0.048, *d* = 0.1, 95% CI [0.02, 4.18]. Additionally, the frequency domain parameter HF was a significantly higher, *t*(30) = 2.71, *p* = 0.01, *d* = 0.17, 95% CI [20.64, 145.89]. No change was observed in LF, *t*(30) = 0.04, *p* = 0.97, *d* = 0.00, 95% CI [−111, 115]. During the nature‐based soundscape, both the mean HR, *t*(50) = −3.06, *p* = 0.004, *d* = 0.43, 95% CI [−2.79, −0.58] and the respiratory rate, *t*(50) = −3.58, *p* < 0.001, *d* = 0.50, 95% CI [−0.02, −0.01] were significantly lower than during the reference soundscape. Expressed in breath per minute, the mean respiratory rate was 13.2 breaths/min (SD = 3.0) during the nature‐based soundscape and 14.4 breaths/min (SD = 3.0) during the reference soundscape. The difference equates to approximately one less breath per minute during the nature‐based soundscape. These findings, characterized by increases in RMSSD and HF along with decreased HR and respiratory rate, suggest enhanced parasympathetic activity. This signifies increased relaxation, as parasympathetic activity is commonly associated with reduced stress and enhanced well‐being. Given the non‐normal nature of the HRV data, the distribution of the parasympathetic parameters is depicted in Figure 3.

**TABLE 1:** The results of the physiological measurements. Based on normality assumptions, variables were analyzed using either a paired *t*‐test (indicated by gray shading) or a robust paired *t*‐test.

	Nature‐based	Reference	Significance	Effect size
	*M* (SD)	*M* (SD)	*p*	*d*
RMSSD (ms)	33.0 (20.9)	30.9 (20.0)	0.048	0.10
HF (ms^2^/Hz)	666 (916)	512 (657)	0.01	0.17
LF (ms^2^/Hz)	863 (1200)	905 (1260)	0.97	0.00
Heart rate (bpm)	69.0 (11.0)	70.7 (10.6)	0.004	0.43
Respiratory rate (Hz)	0.22 (0.05)	0.24 (0.05)	< 0.001	0.50

Abbreviations: HF = high frequency, LF = low frequency, RMSSD = root mean square of successive differences.

### 3.2 Psychological Parameters

The psychological results are presented in Table 2. Upon analyzing participants' responses from Warr's questionnaire, we adopted a four‐factor solution, categorizing self‐reported emotions into anxiety, comfort, depression, and enthusiasm. Both comfort, *t*(32) = 8.64, *p* < 0.001, *d* = 0.86, 95% CI [2.12, 3.43] and enthusiasm, *t*(52) = 5.98, *p* < 0.001, *d* = 0.82, 95% CI [1.07, 2.15] were significantly higher after exposure to the nature‐based soundscape compared to the reference soundscape. This finding suggests that participants not only felt more at ease and relaxed but also experienced heightened positive emotions after listening to the nature‐based soundscape. Moreover, levels of feelings associated with anxiety, *t*(32) = −10.02, *p* < 0.001, *d* = 0.81, 95% CI [−3.63, −2.41], and depression, *t*(32) = 4.16, *p* < 0.001, *d* = 0.34, 95% CI [−1.49, −0.51] were significantly lower after exposure to the nature‐based soundscape. This suggests a reduction in negative emotional states upon exposure to natural sounds.

**TABLE 2:** The results of the psychological measurements. Based on normality assumptions, variables were analyzed using either a paired *t*‐test (indicated by gray shading) or a robust paired *t*‐test. Feelings of anxiety, depression, enthusiasm, and comfort were measured using Warr's scale of affective well‐being. Creativity and belonging were measured using the VAS scale.

	Nature‐based	Reference	Significance	Effect size
	*M* (SD)	*M* (SD)	*p*	*D*
Anxiety	1.8 (1.3)	4.5 (1.9)	< 0.001	0.81
Depression	1.2 (1.4)	2.1 (1.9)	< 0.001	0.34
Enthusiasm	6.9 (1.5)	5.3 (1.7)	< 0.001	0.82
Comfort	8.2 (1.4)	5.6 (2.0)	< 0.001	0.86
Creativity	6.2 (2.1)	4.0 (2.1)	< 0.001	0.72
Belonging	5.8 (2.1)	4.7 (2.5)	< 0.001	0.31

Our data further suggest enhancements in both creativity and feelings of belonging when participants were exposed to the nature‐based soundscape. Specifically, participants reported increased creative feelings, *t*(32) = 7.25, *p* < 0.001, *d* = 0.72, 95% CI [1.83, 3.27]. The finding aligns with previous study showing improved cognitive performance among participants exposed to nature sounds (Van Hedger et al. 2019). These observations support the ART, which posits that natural settings can restore attentional resources. Similarly, feelings of belonging intensified after the nature‐based soundscape exposure, *t*(32) = 2.58, *p* = 0.015, *d* = 0.31, 95% CI [0.26, 2.23]. This increase suggests that natural environments, even when experienced auditorily, can foster a sense of connection. Since humans are inherently part of nature, this connection may extend beyond the individual's relationship with nature to include a broader social context, invoking a deeper sense of belonging.

## 4 Discussion

This study demonstrates comprehensive and consistent well‐being benefits derived from exposure to nature‐based soundscapes in healthy individuals. In line with our hypotheses, we observed a higher RMSSD during exposure to the nature‐based soundscape compared to the reference. Furthermore, our results revealed significant effects on various secondary outcomes. Specifically, we noted lower heart and breathing rate, and higher HF, all of which underscore enhanced parasympathetic activity during exposure to the nature‐based soundscape. Subjective assessments further reinforced these results, with participants reporting reduced feelings associated with anxiety and depression alongside increased sensations of comfort and enthusiasm. Such self‐reports are suggestive of improved affective well‐being, consistent with existing literature (Ratcliffe 2021). These physiological and psychological markers imply that the nature‐based soundscape effectively promoted recovery and relaxation among participants, aligning with the principles of the SRT. Furthermore, after exposure to the nature‐based soundscape, participants also indicated heightened feelings of creativity and a sense of belonging, pointing to enhancements in both cognitive and social well‐being. Taken together, these results further substantiate therapeutic potential of nature‐based soundscapes, highlighting their diverse benefits spanning physiological, emotional, cognitive, and social domains.

### 4.1 Well‐Being Benefits of the Nature‐Based Soundscape

In the present study, an immersive acoustic experience in nature was created by integrating various nature sounds with musical elements. This intervention induced significant effects within a brief 10‐min listening period. Most of the effect sizes for the measured psychological parameters were large, suggesting a substantial impact on participants' psychological well‐being. While the effect sizes for the physiological parameters were from very small to medium, it is noteworthy that the physiological effects were consistently detectable across several parameters even within this short timeframe. This consistent detection of effects is particularly significant given the inherently conservative nature of the robust statistical tests applied to non‐normal data in our analysis. For example, when conventional tests were utilized for the variable ‘creativity’—which was borderline normally distributed (*p* = 0.044)—a Cohen's d value of 1.04 was obtained. In contrast, the robust test, which accounts for the influence of outliers and extreme values, yielded a more conservative effect size of 0.72. It is also reasonable to speculate that the observed physiological effects may accumulate with repeated or prolonged exposure to the nature‐based soundscape.

Interestingly, the health benefits of this soundscape paralleled those of actual nature immersion (Berto 2014; Bratman et al. 2019; Haluza, Schönbauer, and Cervinka 2014; Mygind et al. 2019). This similarity is notable since real nature immersion engages multiple senses, whereas our study solely relied on auditory stimulation. It is possible that the nature‐based soundscape triggered the limbic system's positive biophilic response, subsequently inducing relaxation in the autonomic nervous system (Sudimac, Sale, and Kühn 2022). Furthermore, the act of listening with eyes closed might enable listeners to visualize their personal natural settings. Prior research indicates that the significance of recorded soundscapes lies not just in simulation but also in their ability to stimulate the imagination (Bates et al. 2020; Ratcliffe, Gatersleben, and Sowden 2016). Consequently, the observed benefits may stem from the imaginative reconstruction of nature experiences facilitated by the nature‐based soundscape.

### 4.2 Strengths of the Study

Using a single‐session crossover design allowed us to identify differences in parasympathetic outcomes between the nature‐based and reference soundscapes, consistent with SRT. This design countered the limitations of previous between‐subject designs, plagued by substantial physiological baseline variances and requisite large sample sizes due to the minuteness of measurable changes. The single‐session approach was essential to control for day‐to‐day fluctuations in psychophysiological outcomes like HRV. These fluctuations can be influenced by internal and external stress factors, such as physical activity levels, nutrition, sleeping patterns, illness, and alcohol use. If a participant were to engage in different research conditions on separate days, these factors could distort the results (Laborde, Mosley, and Thayer 2017). Additional strength of our study was the comparative analysis of nature‐based soundscape against a reference condition, exemplifying respite soundscapes (Ferreira, Ferreira, and Bos 2021). The observed differences between the soundscapes highlighted the enhanced and specific relaxation advantages and the profundity of relaxation attainable through nature‐based soundscapes.

### 4.3 Limitations and Future Direction

While the observed differences between the soundscapes are notable, there was inter‐individual variability in physiological responses. These variations might be attributed to participants' inconsistent ability to relax or their varying degrees of connectedness with nature. In this study, we estimated respiratory rate from RR data (Tarvainen et al. 2014). However, deriving respiratory rate from heart rate data presents certain challenges. Using HRV as an indirect measure of respiratory rate may not fully capture the nuances of breathing patterns. While this method is effective for estimating respiratory rate, it may not be as precise as direct respiratory measurements. Additionally, there is the potential for statistical overlap between HRV and estimated respiratory rate. This could lead to redundancy in statistical models, potentially complicating the interpretation of their independent effects. While the Kubios software provides a reliable estimation method, future studies might benefit from direct respiratory measurements to increase precision.

This study was limited to examining short‐term effects, underscoring the need for future investigations into the long‐term impacts of nature‐based soundscapes. Increasing parasympathetic activity acutely can trigger a chain of beneficial physiological changes potentially alleviating short‐ or long‐term stress responses (Schneiderman et al. 2005). Thus, nature‐based soundscapes could significantly enhance daily stress management and promote long‐term recovery beyond the immediate listening experience, meriting longer term experimental investigation.

Additionally, this study focused on healthy adults, suggesting a need for future research on clinical populations. When assessing clinical populations, it would be valuable to also measure outcomes such as the length of hospital stays and medication requirements. While the purpose of this study was to compare relaxation between natural and urban environments, we acknowledge that adding a silence condition could provide valuable insights. In modern everyday life, it is increasingly difficult to find silence for relaxation, and many people do not find it natural or comfortable to rest in complete silence. However, silence may also be effective in promoting relaxation and should be investigated in future studies.

### 4.4 Applicability

Utilizing mobile applications that incorporate nature‐based soundscapes would enable easy, ongoing access to these therapeutic auditory experiences, potentially benefiting a broad audience and offering a means for prevention. The health benefits of these soundscapes were observed in a relatively short period, suggesting their utility in various everyday settings. Individuals could use these soundscapes during breaks at work, in educational environments, during commutes, or in any situation where relaxation and mental decompression are needed. This is especially significant for those who have limited access to real nature. By simulating the serene and restorative ambiance of nature, these digital soundscapes can offer a valuable mental health resource, bridging the gap for people living in urban or built‐up areas where real nature is less accessible. Thus, nature‐based soundscapes emerge not only as a tool for immediate stress relief but also as a strategic element in promoting long‐term mental health and well‐being across various populations.

## 5 Conclusion

In summary, the heightened HRV and reduced respiratory rate observed during the nature‐based soundscape sessions show enhanced relaxation and physiological benefits in healthy individuals. Furthermore, improvements in affective, cognitive and social well‐being were noted when compared to the reference condition. These encouraging findings emphasize the therapeutic potential of nature‐based soundscapes for health and recovery and present need for extended research. Further exploration into the long‐term effects and broader applicability of these soundscapes is essential, particularly for diverse populations grappling with mental health challenges. This research could have far‐reaching implications, potentially informing future studies, policy‐making, and therapeutic practices in mental health care.

## Author Contributions

**Susanne Kumpulainen:** conceptualization, data curation, funding acquisition, methodology, project administration, software, supervision, visualization, writing – original draft. **Samad Esmaeilzadeh:** data curation, formal analysis, investigation, software, writing – review and editing. **Markus Pesonen:** conceptualization, methodology, writing – review and editing. **Catarina Brazão:** conceptualization, methodology, writing – review and editing. **Arto J. Pesola:** conceptualization, funding acquisition, resources, supervision, writing – review and editing.

## Conflicts of Interest

Markus Pesonen and Catarina Brazão are founders of Olo Space and have designed the nature‐based sound journey as part of their product, which was also used in the present study. We confirm that there is no other conflict of interest associated with this research and no external factors influenced the objectivity of our findings. We have fully disclosed all pertinent commercial and other relationships, as per the journal's conflict of interest policy, and affirm that the results presented in this manuscript are unbiased and based solely on the scientific merits of the study.

## Data Availability

The authors confirm that the data supporting the findings of this study are available within the article.
